# Draft genome of a human-derived *pks+ E. coli* that caused spontaneous disseminated infection in a mouse

**DOI:** 10.1128/mra.00387-24

**Published:** 2024-06-04

**Authors:** Allison M. Weis, O’Connor J. Matthews, Matthew A. Mulvey, June L. Round

**Affiliations:** 1 Department of Pathology, Division of Microbiology and Immunology, University of Utah School of Medicine, Salt Lake City, Utah, USA; 2 Huntsman Cancer Institute, University of Utah, Salt Lake City, Utah, USA; 3 School of Biological Sciences, University of Utah, Salt Lake City, Utah, USA; 4 Henry Eyring Center for Cell & Genome Science, University of Utah, Salt Lake City, Utah, USA; Department of Biological Sciences, Wellesley College, Wellesley, Massachusetts, USA

**Keywords:** *E. coli*, gastrointestinal tract, genome, pathogen

## Abstract

We present the draft genome of a novel human-derived *Escherichia coli* strain isolated from a healthy control human microbiota that, when put into a mouse, spontaneously disseminated from the gut to the kidneys.

## ANNOUNCEMENT

*Escherichia coli* is a common gut commensal as well as a devastating and persistent pathogen. Pathogenic strains of *E. coli* can cause myriad types of infections, including diarrhea, urinary tract infections, bacteremia, and sepsis (1–3). Furthermore, *E. coli* strains that carry the *pks*+ gene cluster, encoding the secreted DNA-damaging toxin colibactin, can promote tumor development and are associated with colorectal cancer (4–6). Here, we present the draft genome of a novel human-derived strain isolated from a mouse after spontaneous dissemination from the gut.

This *E. coli* strain was recovered from the kidneys of an ex-germ-free Swiss Webster mouse, raised in house, which had undergone a human-to-mouse fecal microbial transplant in the Round lab at the University of Utah, IACUC protocol 00001562. The human sample was from the ColoCare study (7). The mouse was discovered near death, and upon necropsy, both kidneys were red and enlarged. Kidney homogenates were plated on LB agar and were incubated aerobically at 37°C overnight. The kidneys contained numerous bacteria, all seemingly the same type, which were then streaked to isolation. Plating on McConkey agar led to brilliant pink hues of the colonies, indicative of *E. coli*. We named the isolate AW001.

DNA was extracted from a pure culture of AW001 grown overnight in LB broth at 37°C in the Mulvey lab using the Qiagen DNeasy Blood & Tissue kit, and libraries were made using Tecan Ultralow V2, both according to the manufacturer protocols. Sequencing was performed by Illumina NovaSEQ6000 at the University of Colorado Anschutz with 151 bp paired-end reads. The total read count was 8,779,520, with an average depth of 257.79. Software default parameters were used, except where noted. Read preprocessing was performed using Trim Galore v0.6.5dev (8). Sequences were assembled *de novo* using Unicycler v.0.4.8 through BV-BRC v3.35.5 using -t 12 –min_fasta_length 300 –keep 2 –no_pilon (9–11). The assembled genome contains 109 contigs with an N50 value of 268,981. The genome was annotated using Genbank PGAP v6.6 (12). CheckM v1.0.5 revealed completeness of 99.97 (13).

The assembled AW001 genome contains 5,008,932 bps, comprising 4,952 genes, 4,868 CDS, and 50.7% GC content. AW001 was predicted to be *E. coli* phylogroup B2 using ClermonTyping (v23.06) (14). By MLST, it was ST “unknown,” closely related STs being 12998 and 2831 (MLST-2.0 Server). Using AMRFinderPlus v3.11.26 (15), putative antimicrobial resistance genes were identified including fosfomycin-resistant *glpT* variant (16), multidrug-resistance *marR*, colistin-resistant *pmrB* (17), efflux pump acrF (18), and beta-lactamase *blaEC* (19) (Table 1). This indicates that AW001 is likely resistant to multiple classes of clinically relevant antibiotics.

**TABLE 1:** Key AMR and virulence factors identified in the AW001 *E. coli* genome

Gene(s)	Protein ID	Type	Protein name and function
*acrF*	WP_001273251.1	AMR	AcrF multidrug efflux RND transporter permease
*blaEC*	WP_001556381.1	AMR	BlaEC family class C beta-lactamase
*glpT*	WP_000948732.1	AMR	GlpT fosfomycin resistant
*marR*	WP_000799375.1	AMR	MarR multidrug resistant
*pmrB*	WP_001052123.1	AMR	PmrB colistin resistant
*ariR*	WP_000888771.1	Stress	Biofilm/acid-resistance regulator AriR
*emrE*	WP_001070440.1	Stress	EmrE multidrug efflux SMR transporter
*astA*	WP_000989438.1	Virulence	EAST1 heat-stable enterotoxin
*cbtA*	WP_000854814.1	Virulence	CbtA type IV toxin-antitoxin system
*ccdA*	WP_000125566.1	Virulence	CcdA type II toxin-antitoxin system CcdA
*chuA*	WP_000089583.1	Virulence	ChuA outer membrane hemin receptor
*ClbA*	WP_001217110.1	Virulence	Colibactin synthesis proteins (*pks*)
*entH*	WP_000637953.1	Virulence	EntH proofreading thioesterase
*fdeC*	WP_000092543.1	Virulence	FdeC inverse autotransporter adhesin
*fimH*	WP_000832236.1	Virulence	SfaH fimbrial protein subunit
*fliP*	WP_334615852.1	Virulence	FliP flagellar type III secretion system
*gspA*	WP_000107592.1	Virulence	GspA,C,D type II secretion system
*gspG*	WP_001087296.1	Virulence	GspG type II secretion system major pseudopilin
*hcp*	WP_000458845.1	Virulence	Hcp family type VI secretion system effector
*hecB*	WP_334616364.1	Virulence	HecB family hemolysin secretion/activation protein
*hhA*	WP_001333231.1	Virulence	Hha hemolysin expression modulator
*ibsE*	WP_001387082.1	Virulence	Ibs family toxin type I toxin-antitoxin system
*Irp1,2*	WP_000369530.1	Virulence	Yersiniabactin siderophore
*iss*	WP_001298464.1	Virulence	Iss increased serum survival lipoprotein
*ldrD*	WP_001295224.1	Virulence	Ldr family protein type I toxin-antitoxin system
*mchB*	WP_001375214.1	Virulence	H47 microcin
*mchF*	WP_001518504.1	Virulence	MchF microcin H47 export transporter peptidase
*neuC*	WP_000723250.1	Virulence	Polysialic acid biosynthesis protein P7
*ompA*	WP_001518466.1	Virulence	Outer membrane protein
*paeA*	WP_000935036.1	Virulence	Hemolysin family protein
*sitC*	WP_001101732.1	Virulence	MntB manganese transport membrane protein
*sslE*	WP_001034565.1	Virulence	SslE lipoprotein metalloprotease
*TssE*	WP_000106967.1	Virulence	TssE type VI secretion system baseplate subunit
*tssJ*	WP_000484008.1	Virulence	TssJ type VI secretion system lipoprotein
*vgrG*	WP_001350146.1	Virulence	VgrG type VI secretion system tip protein
*ybtE*	WP_001518699.1	Virulence	2,3-dihydroxybenzoate-AMP ligase
*ybtP*	WP_001327262.1	Virulence	YbtP yersiniabactin ABC transporter
*ybtQ*	WP_001295637.1	Virulence	YbtQ yersiniabactin ABC transporter
*yqfA*	WP_000250274.1	Virulence	Hemolysin III family protein

AW001 was analyzed for putative fitness and virulence factors using AMRFinderPlus, BLAST+ (v2.14.0+), and targeted searching (20). Like *E. coli* strain NC101 (21), AW001 carries the *clb (pks*) gene cluster that produces colibactin. AW001 contains genes involved in iron acquisition, adhesion, capsule biosynthesis, and mucin degradation (Table 1). AW001 also encodes toxin-antitoxin systems; enterotoxin AstA; antimicrobial microcin H47; type II, III, and VI secretion systems; and hemolysin biosynthesis genes (Table 1).

## Data Availability

Data are in GenBank under accession JAYWIW000000000. Raw reads are under SRR28249370.
